# Supplementary material for: Revealing the mechanisms of membrane protein export by virulence-associated bacterial secretion systems
Source: Nat Commun. 2018 Aug 27;9:3467. doi: 10.1038/s41467-018-05969-w (PMC6110835; doi:10.1038/s41467-018-05969-w)
Supplement: Supplementary file 1 — Supplementary Information [file 41467_2018_5969_MOESM1_ESM.pdf]

Supplementary Information to the manuscript „Revealing the mechanisms of membrane protein export by virulence-associated bacterial secretion systems" by Krampen et al.

Supplementary Figures 1-16 are provided in this PDF document.

Supplementary Data 1-3 are provided separately as .xlsx files.

Supplementary Data 1: ΔG prediction of T3SS and T4BSS TMD-containing substrates for membrane partitioning (window 18-35, length correction ON) and SRP binding (window 12-17 length correction OFF)

Supplementary Data 2: List of proteins identified by mass spectrometry in $SseF_{V73pBpa}$ crosslinked band and uncrosslinked control

Supplementary Data 3: Strains, plasmids, and primers

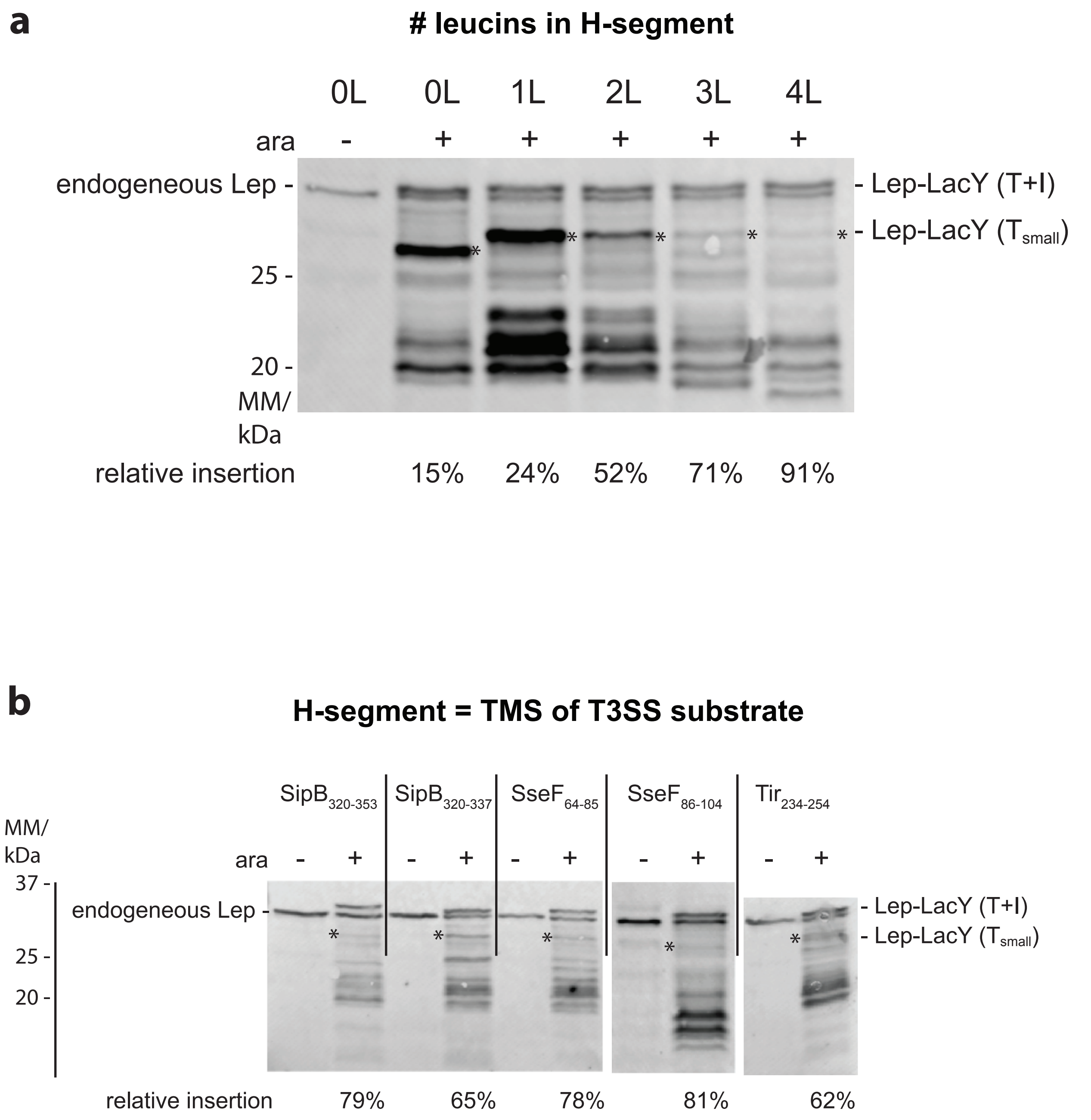

Supplementary Figure 1: Transmembrane segment insertion assay for standard Lep-LacY constructs

a Lep-LacY constructs with different amounts of leucins (0L-4L) were expressed in *E. coli* BW25113 and used as a control for the TMS insertion assay. Bacteria were grown to OD600 = 0.4 and protein expression was induced by addition of arabinose to a final concentration of 0.2%. Samples were analyzed by SDS PAGE, Western blotting and immunodetection of the Lep P2 domain. A representative result of three independent experiments is shown. The ratio of uncleaved (upper band) to cleaved (asterisk) Lep-LacY was calculated and plotted in the diagram. **b** Uncropped image of Western blot shown in **Fig. 2c**. Abbreviations: Lep: leader peptidase; Lep-LacY (T+I): translocated and integrated membrane helix; Lep-LacY (T_{small}): small translocated form of Lep-LacY.

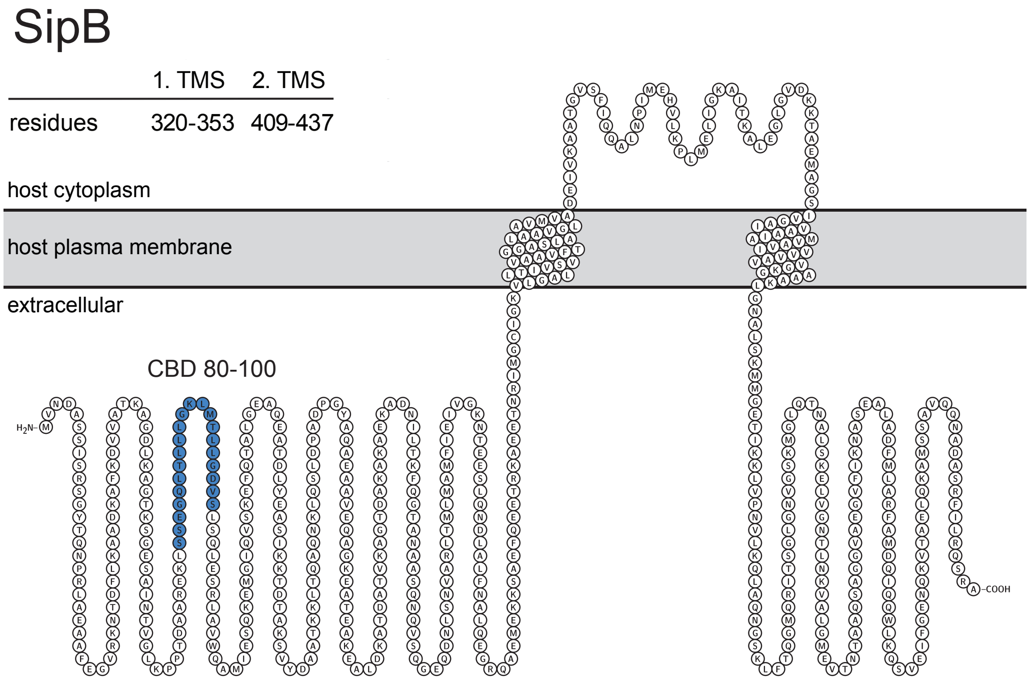

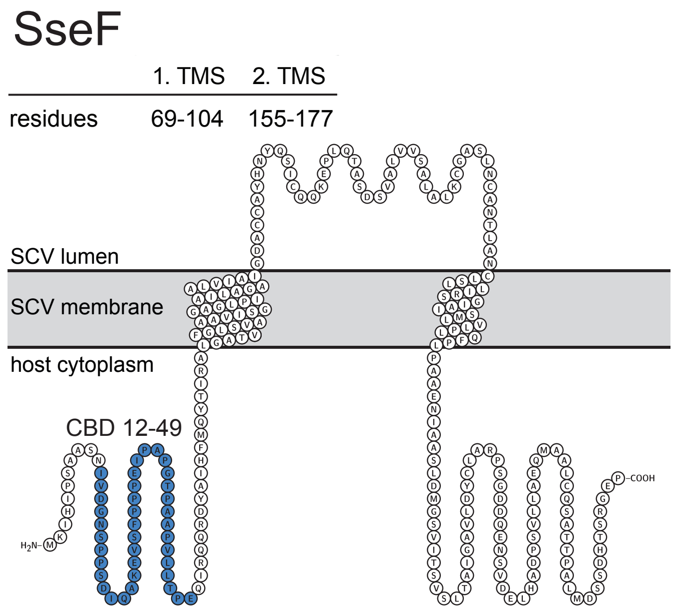

Supplementary Figure 2: Type III-secreted transmembrane proteins and their predicted topology.
Protter[1] visualization of SipB and SseF from Salmonella and Tir from EPEC presenting predicted topologies of their TMDs. The locations of the TMS was predicted using Δ Gpred in the full protein scan mode as described in the Materials and Methods section. Chaperone binding domains (CBD) are indicated as reported (blue)[2-4]. Abbreviations: CBD: chaperone binding domain; TMS: transmembrane segment.

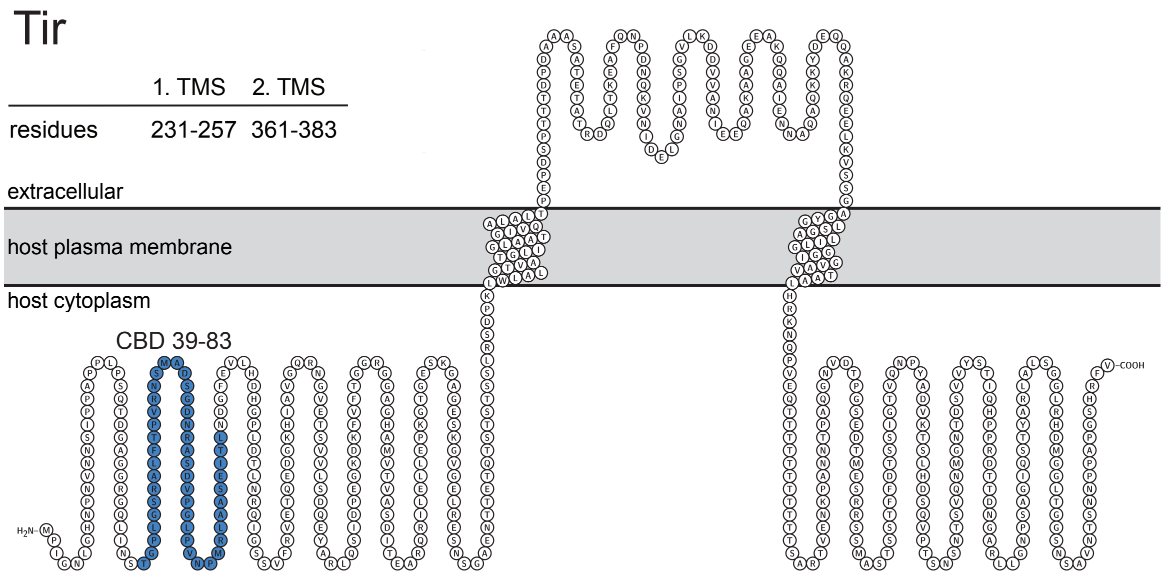

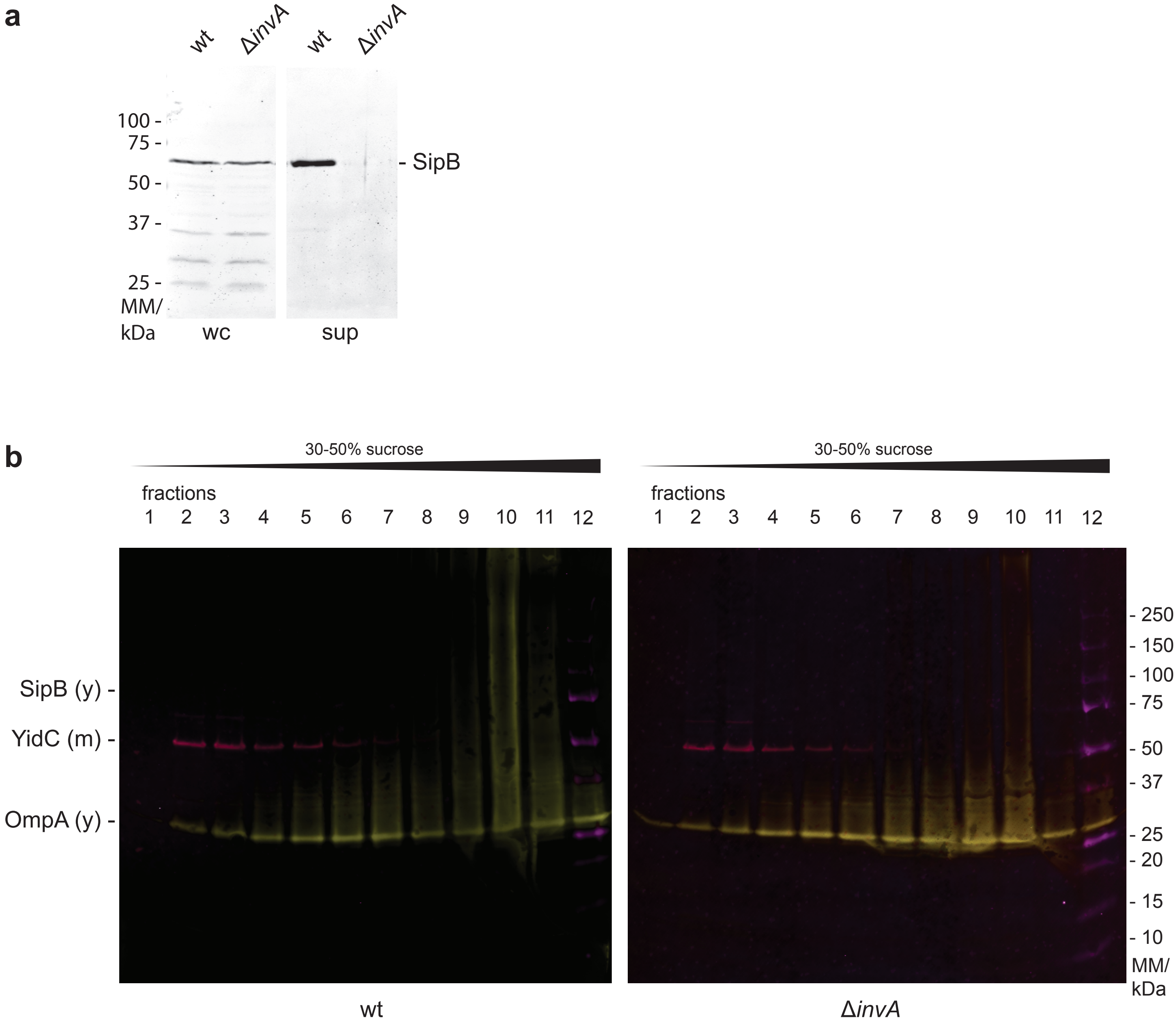

Supplementary Figure 3: Secretion and subcellular localization of SipB in *Salmonella* Typhimurium.
a Expression levels of SipB in *Salmonella* wild type (wt) and type III-secretion deficient mutant (Δ*invA*) strains were analyzed in whole cell lysates (wc) by SDS PAGE, Western blotting and immunodetection. Secretion levels of SipB were assayed by SDS PAGE of TCA precipitated culture supernatants (sup) followed by Western blotting and immunodetection. **b** To assay the propensity of SipB to integrate into the bacterial inner membrane, *Salmonella* wt and Δ*invA* were grown for 5 hours and SipB was analyzed by SDS PAGE and Western blotting of bacterial membrane fractions separated by sucrose-gradient centrifugation. Immunodetection was done with anti-SipB, anti-YidC (inner membrane control) and anti-OmpA (outer membrane control) antibodies. SipB was not detectable dispite of a strong antibody (yellow channel). YidC is shown in the magenta channel, OmpA in the yellow channel. Representative results of three independent experiments are shown. Abbreviations: sup: supernatant; wc: whole cell lysates.

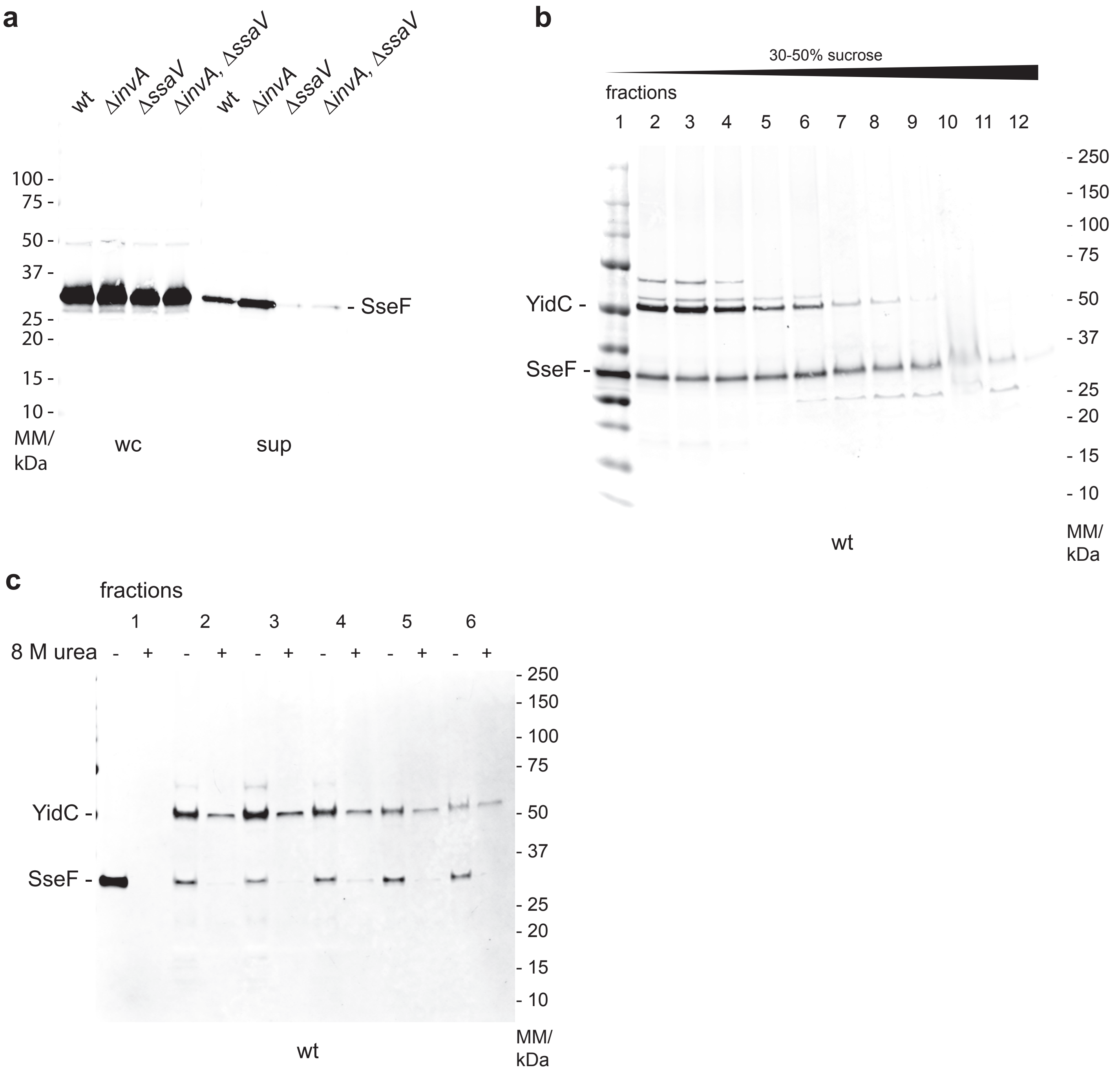

Supplementary Figure 4: Secretion and subcellular localization of SseF in *Salmonella* Typhimurium.
a Expression levels of 3xFLAG tagged SseF in the *Salmonella* wild type (wt) and indicated type III-secretion deficient mutant (T3SS-1: Δ*invA,* T3SS-2: Δ*sscV*) strains were analyzed in whole cell lysates (wc) by SDS PAGE, Western blotting and immunodetection. Secretion levels of 3xFLAG tagged SseF were assayed by SDS PAGE of TCA precipitated culture supernatants (sup) followed by Western blotting and immunodetection. **b** Immunodetection of SseF^{FLAG} in the *Salmonella* wt in bacterial membrane fractions across a sucrose-gradient after SDS PAGE and Western blotting. Fraction 1 also contains the molecular mass standard. YidC serves as an inner membrane control. **c** Immunodetection of SseF^{FLAG} and YidC in bacterial inner membrane fractions (#1-6) treated with and without 8 M urea. Representative results of three independent experiments are shown. Abbreviations: sup: supernatant; wc: whole cell lysates.

a

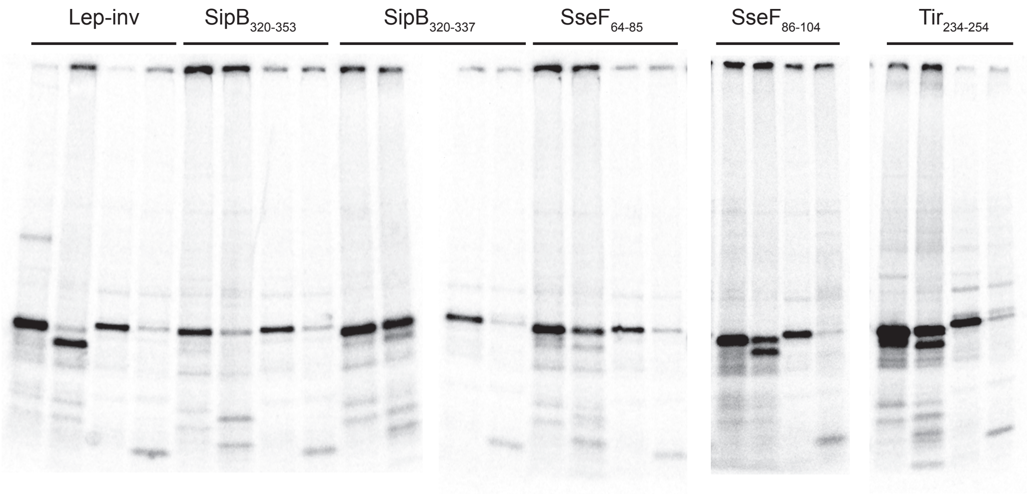

b

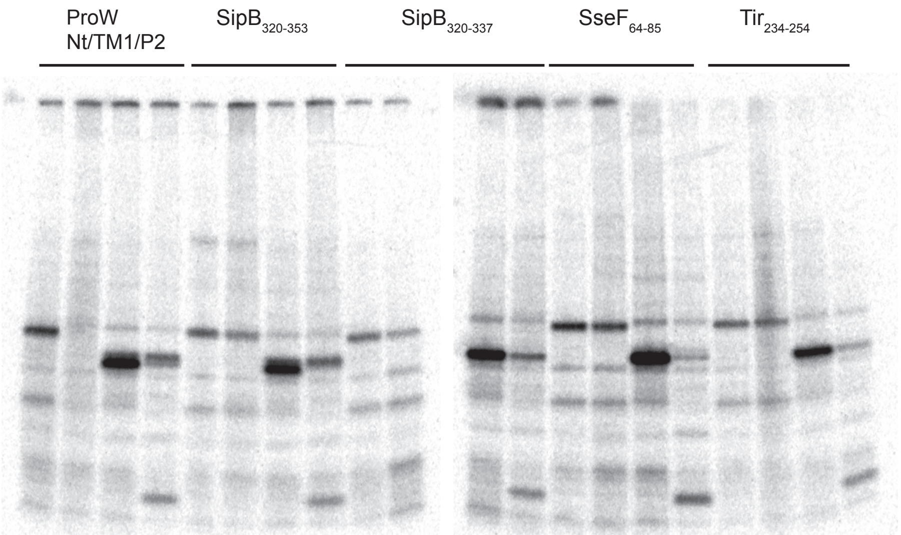

Supplementary Figure 5: Uncropped images of autoradiographs shown in Fig. 3

a

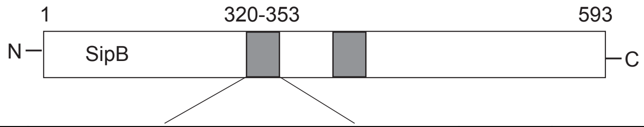

name	sequence (1. ΔG_{app}^{SRP} minimum; 2. ΔG_{app}^{SRP} minimum)	ΔG_{app}^{SRP} - window 12-17 - length correction OFF: 1. ΔG_{app}^{SRP} min.	2. ΔG_{app}^{SRP} min.
SipB wt	VLGALLTIVSVVAAVFTGGASLALAAVGLAVMVA	-0.342	-0.513
A332L	VLGALLTIVSVVLAVFTGGASLALAAVGLAVMVA	-0.837	-0.513
T336L	VLGALLTIVSVVAAVFLGGASLALAAVGLAVMVA	-0.776	-0.513
T326L	VLGALLLIVSVVAAVFTGGASLALAAVGLAVMVA	-1.115	-0.513
G337L	VLGALLTIVSVVAAVFTLGASLALAAVGLAVMVA	-0.639	-0.513
G322L	VLLALLTIVSVVAAVFTGGASLALAAVGLAVMVA	-0.915	-0,513
S329L	VLGALLTIVLVVAAVFTGGASLALAAVGLAVMVA	-1.708	-0.513
G322L,G337L	VLLALLTIVSVVAAVFTLGASLALAAVGLAVMVA	-1.361	-0.513
T326L,S329L	VLGALLLIVLVVAAVFTGGASLALAAVGLAVMVA	-2.511	-0.513
Lep-TMD	ALILVIATLVTGILWCV	-1.119	

low → hydrophobicity → high

b

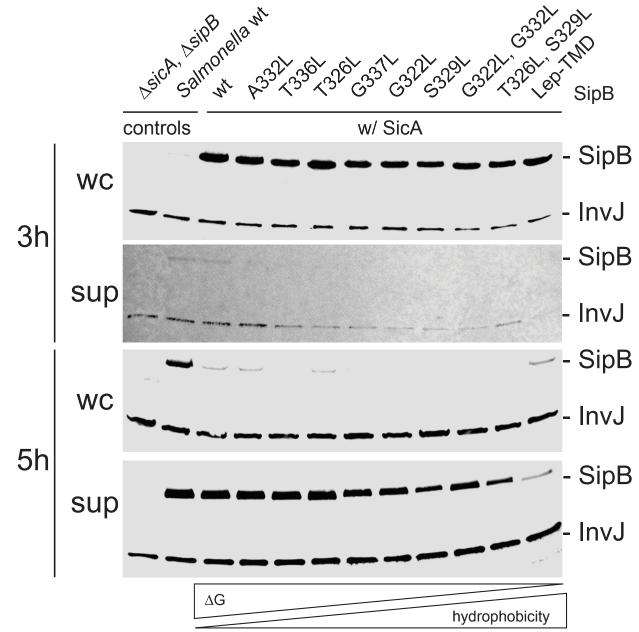

c

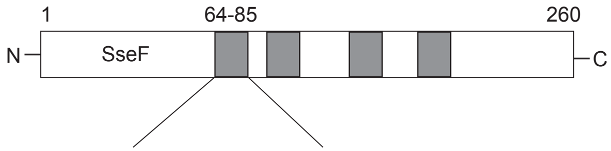

name	sequence (ΔG_{app}^{SRP} minimum)	ΔG_{app}^{SRP} - window 12-17 - length correction OFF
SseF wt	YTIRALGATVVFGLSVAAAVIS	0.467
F75L	YTIRALGATVVLGLSVAAAVIS	0.307
R67L	YTILALGATVVFGLSVAAAVIS	-0.328
T72L	YTIRALGALVVFGLSVAAAVIS	-0.429
T72L,F75L	YTIRALGALVVLGLSVAAAVIS	-0.556
R67L,T72L	YTILALGALVVFGLSVAAAVIS	-1.128
R67L,S78L	YTILALGATVVFGLLVAAAVIS	-0.522
R67L,T72L,S78L	YTILALGALVVFGLLVAAAVIS	-1.65

low → hydrophobicity → high

d

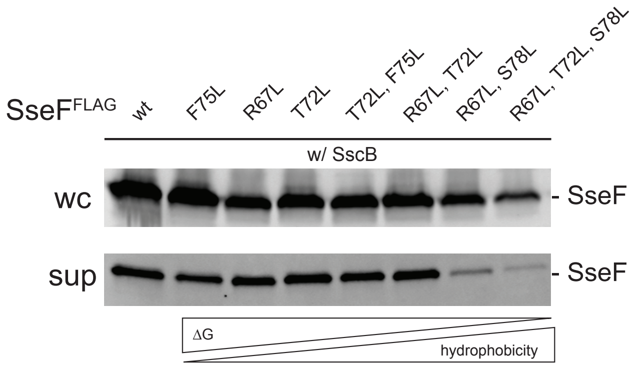

Supplementary Figure 6: Expression and type III-dependent secretion of SipB and SseF mutants with increased hydrophobicity of their first TMS.
a Sequence of the first predicted TMS of SipB indicating stretches of lowest ΔG_{app} for windows of 12-17 aa (SRP-binding windows, ΔG_{app}^{SRP}). Values of the two calculated ΔG_{app}^{SRP} minima are shown on the right. Positions within the first ΔG_{app}^{SRP} minimum were mutated to leucines as indicated (red) to obtain lower ΔG_{app}^{SRP} values. In addition a chimera was constructed, in which the first TMS of SipB was replaced by the first TMS of leader peptidase (Lep). **b** Expression levels of SipB constructs with increasing hydrophobicity were analyzed in whole cell lysates (wc) of *Salmonella* Δ*sicA*, Δ*sipB* mutant strains after 3 and 5 hours of culture, respectively, by SDS PAGE, Western blotting and immunodetection of SipB. Secretion levels of SipB were assayed by SDS PAGE of TCA precipitated culture supernatants (sup) followed by Western blotting. Immunodetection was done with anti-SipB and anti-InvJ (secretion control). **a** As in **a** but showing TMS sequences and ΔG_{app}^{SRP} predictions of SseF constructs. **d.** Expression levels of 3xFLAG tagged SseF constructs with increasing hydrophobicity were analyzed in whole cell lysates (wc) and in TCA precipitated culture supernatants (sup) by SDS PAGE, Western blotting and immunodetection. Representative results of three independent experiments are shown. Abbreviations: sup: culture supernatant; wc: whole cell lysates.

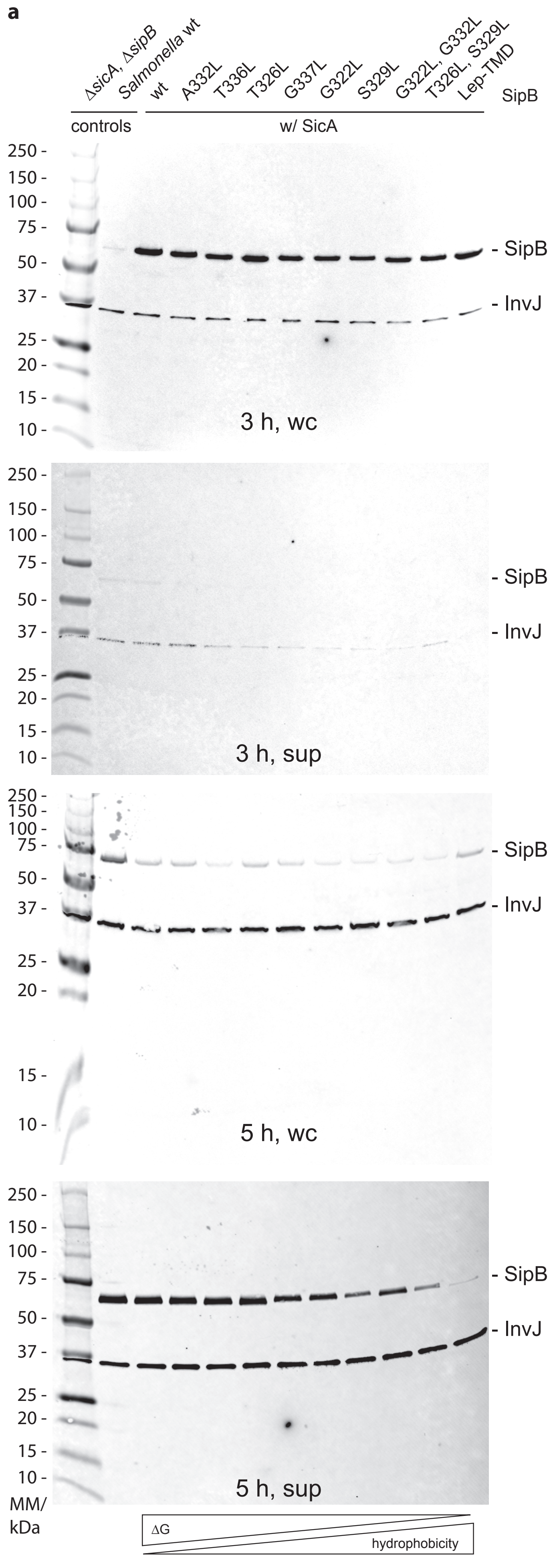

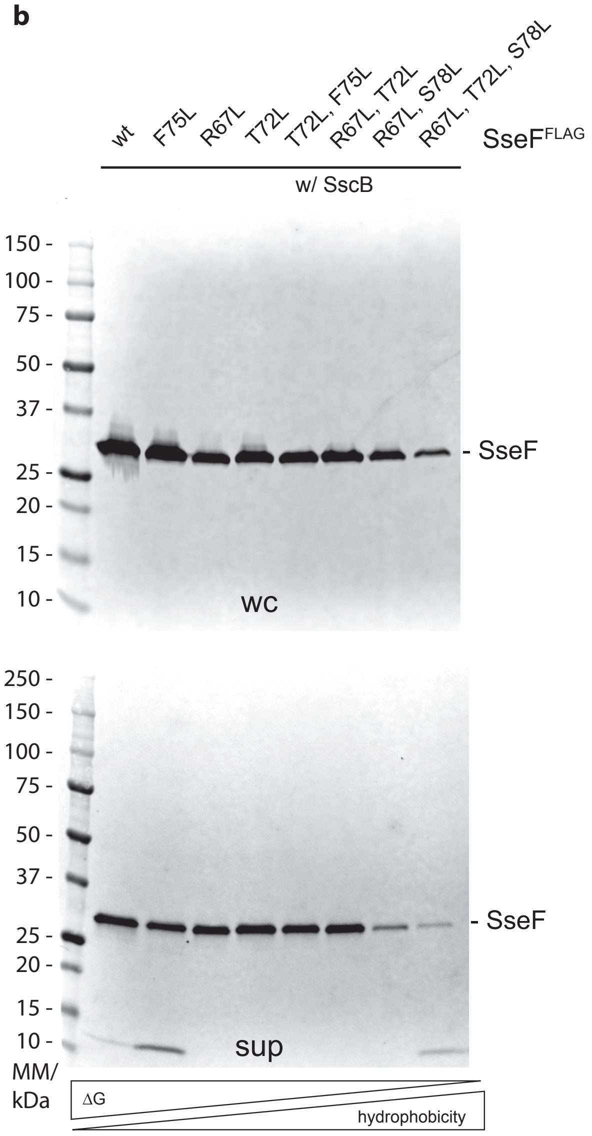

Supplementary Figure 7: Uncropped images of Western blots shown in Supplementary Fig. 6

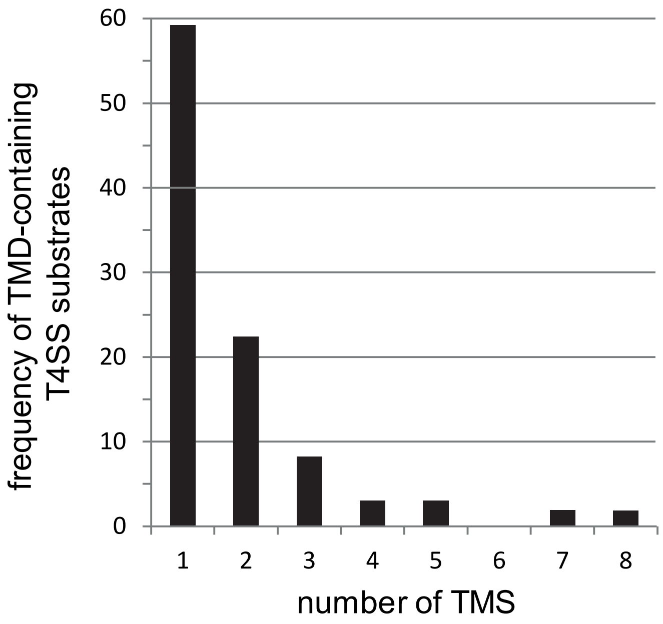

Supplementary Figure 8: Type IVB-secreted transmembrane proteins
T4BSS substrates were analyzed using the full protein scan of the ΔG predictor (http://dgpred.cbr.su.se/, windows: 18-35 aa, length correction: OFF). The histogram shows the distribution of the number of TMS of type III-secreted transmembrane proteins. Abbreviations: TMD: transmembrane domain; TMS: transmembrane segment.

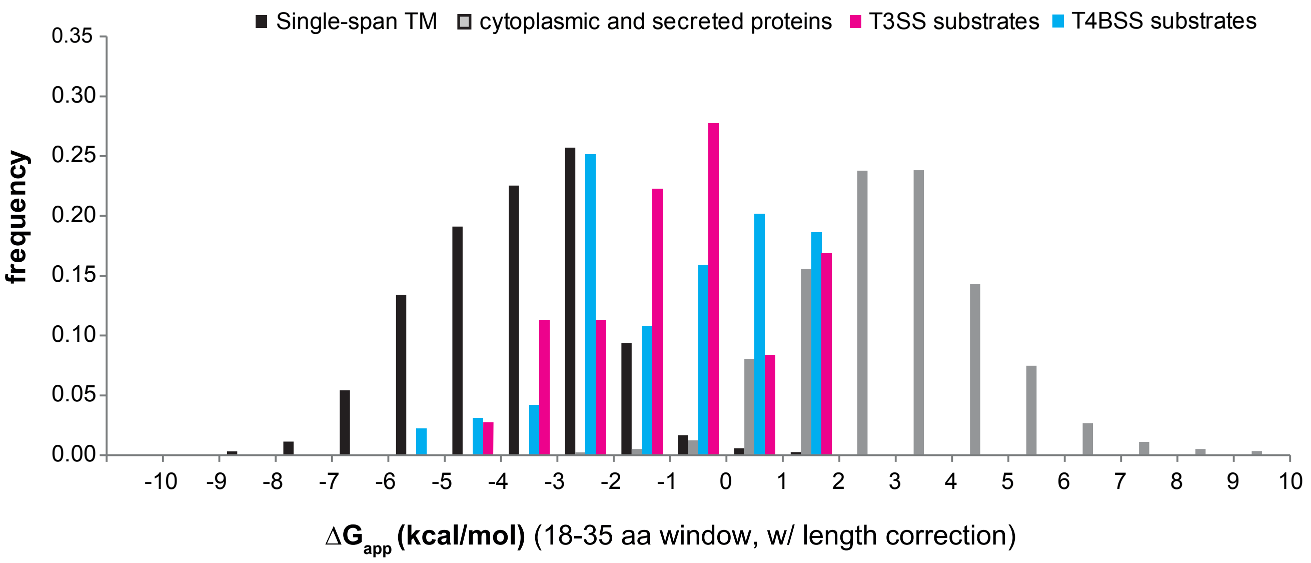

Supplementary Figure 9: Most negative TMSs of T4BSS substrates are less hydrophobic comparing to single-span transmembrane proteins

Distribution of the predicted ΔG_{app} of TMS of T3SS substrates (red) and T4BSS substrates (blue) compared to previously published values for ΔG_{app} of regular transmembrane- and soluble proteins, respectively[5]. For each protein, only its lowest ΔG_{app} of any given sequence window is shown (ΔG predictor settings: window size: 18-35 aa, length correction: ON). Abbreviations: TMS: transmembrane segments.

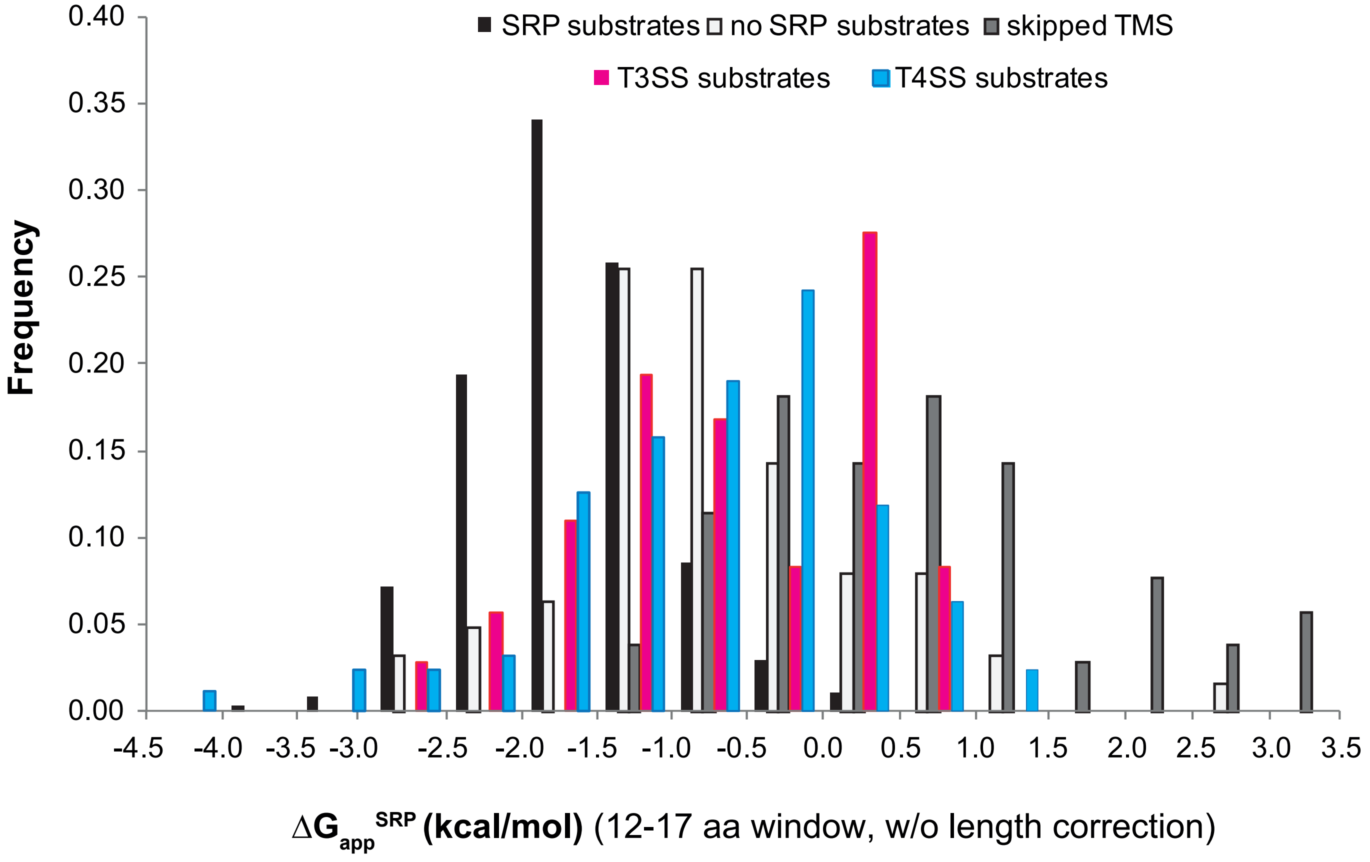

Supplementary Figure 10: Potential of T4BSS substrates to be targeted by SRP
Prediction of ΔG_{app} for the SRP-targeting window of 12-17 aa for transmembrane proteins of type IV B-secreted transmembrane proteins (blue) compared to type III-secreted transmembrane proteins (red) and *E. coli* membrane proteins. The classification of *E. coli* membrane proteins is according to Schibich et al.[6]: SRP substrates (dark grey), non SRP substrates (middle grey) and substrates, in which the first TMS was skipped by SRP (light grey).

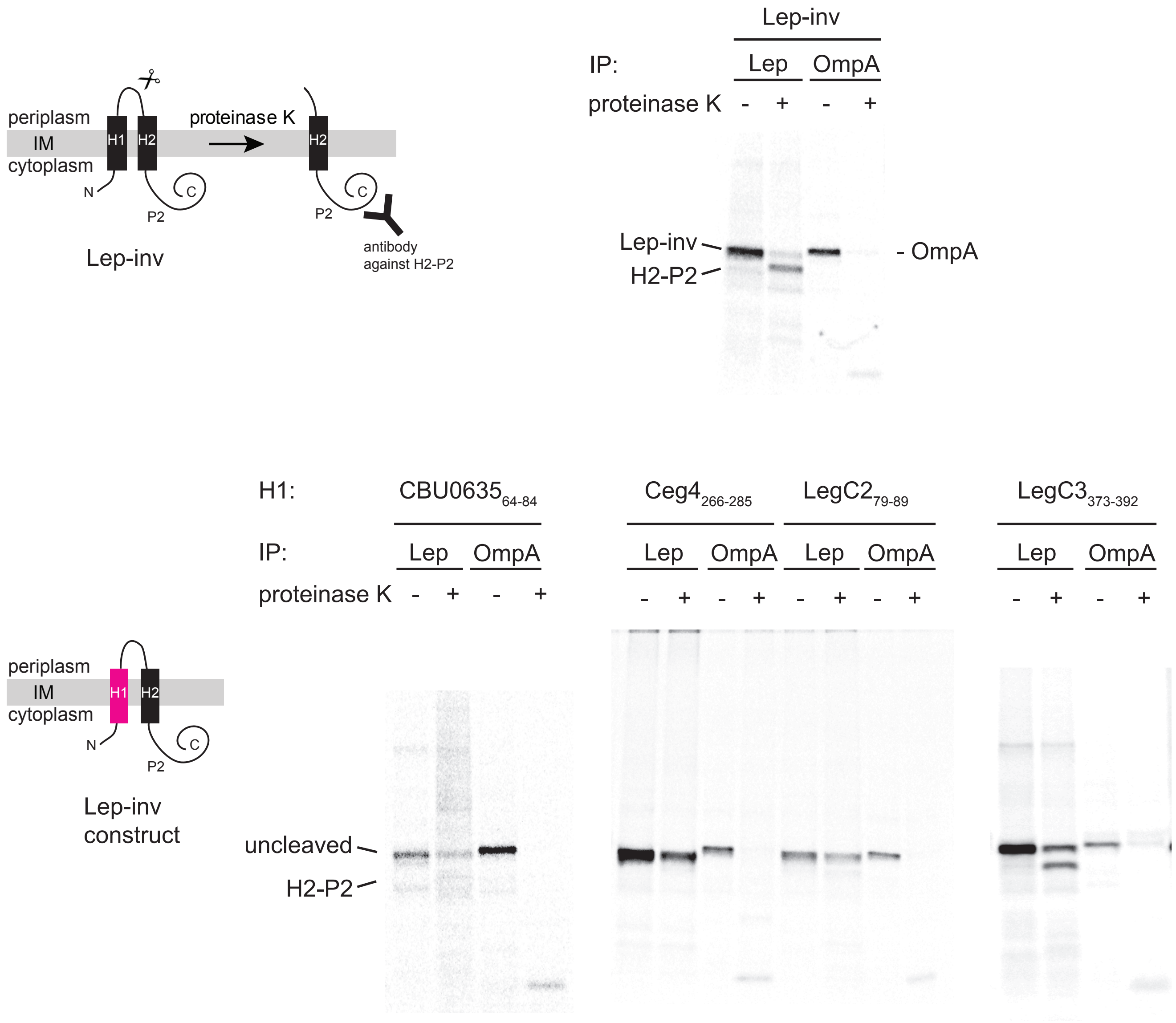

Supplementary Figure 11: Experimental validation of membrane targeting potential of T4BSS substrates
Relevant TMS of T4BSS substrates were assessed for their inner membrane targeting potential and membrane integration in a S-35 Met-based pulse-chase targeting assay using inverted leader-peptidase (Lep-inv)[7]. The principle of the assay is shown on the left. Membrane-inserted Lep-inv was cleaved into a smaller fragment by exogenously added proteinase K. Lep-inv that fails to insert into the membrane is not affected by proteinase K. Cleavage can be detected by immunoprecipitation. For assessment of targeting, the H1 segment of Lep-inv was exchanged against the indicated segment of interest. Proteins were expressed in *E. coli* MC4100 from rhamnose-inducible plasmids. After spheroplasting and addition of proteinase K, proteins of interest were immunoprecipitated and analyzed by SDS PAGE and autoradiography of 35-S. The outer membrane protein OmpA served as control for successful proteinase K digestion. A representative result of three independent experiments is shown. Abbreviations: IM: inner membrane.

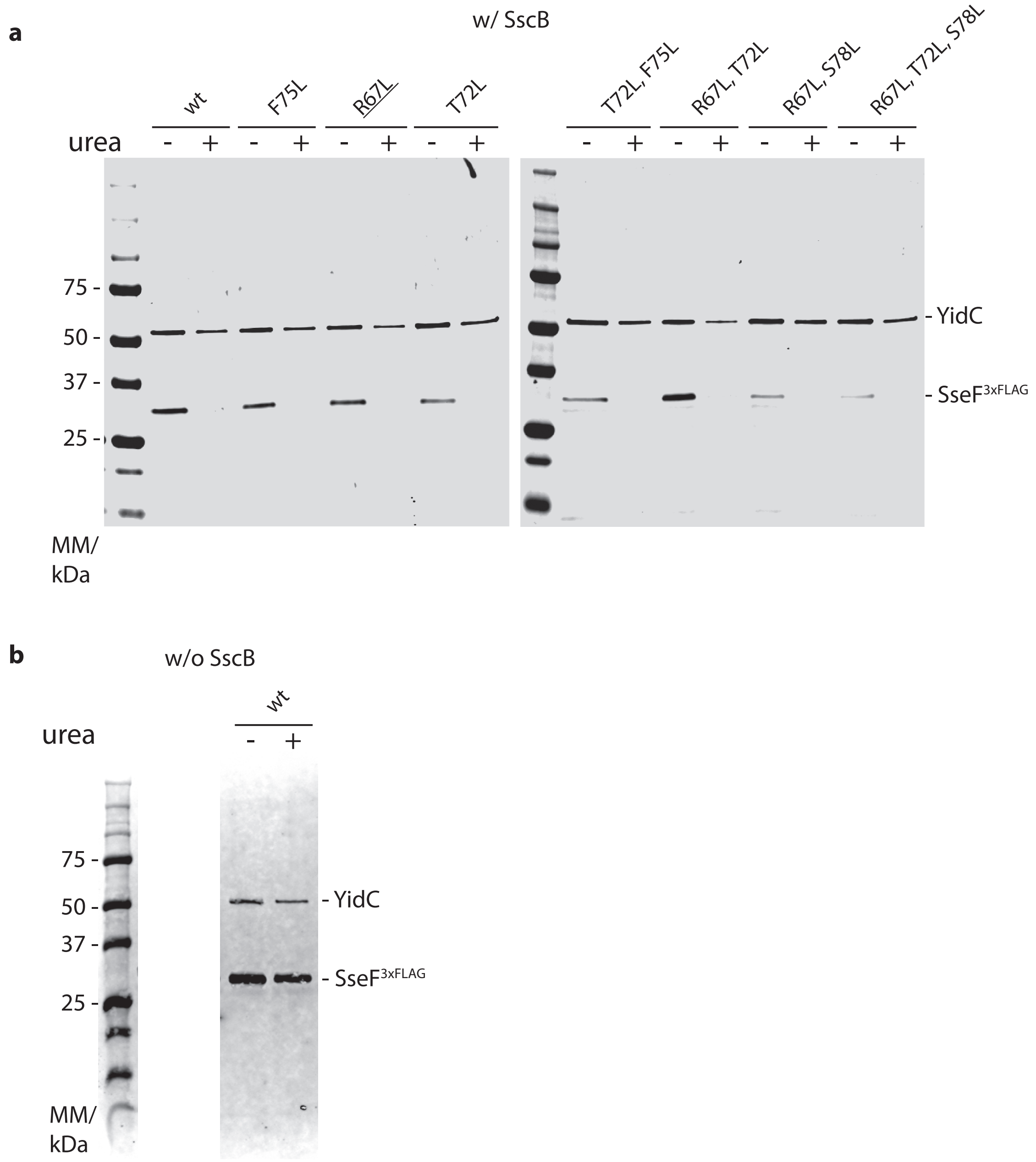

Supplementary Figure 12: Uncropped images of the Western blots shown in Fig. 4

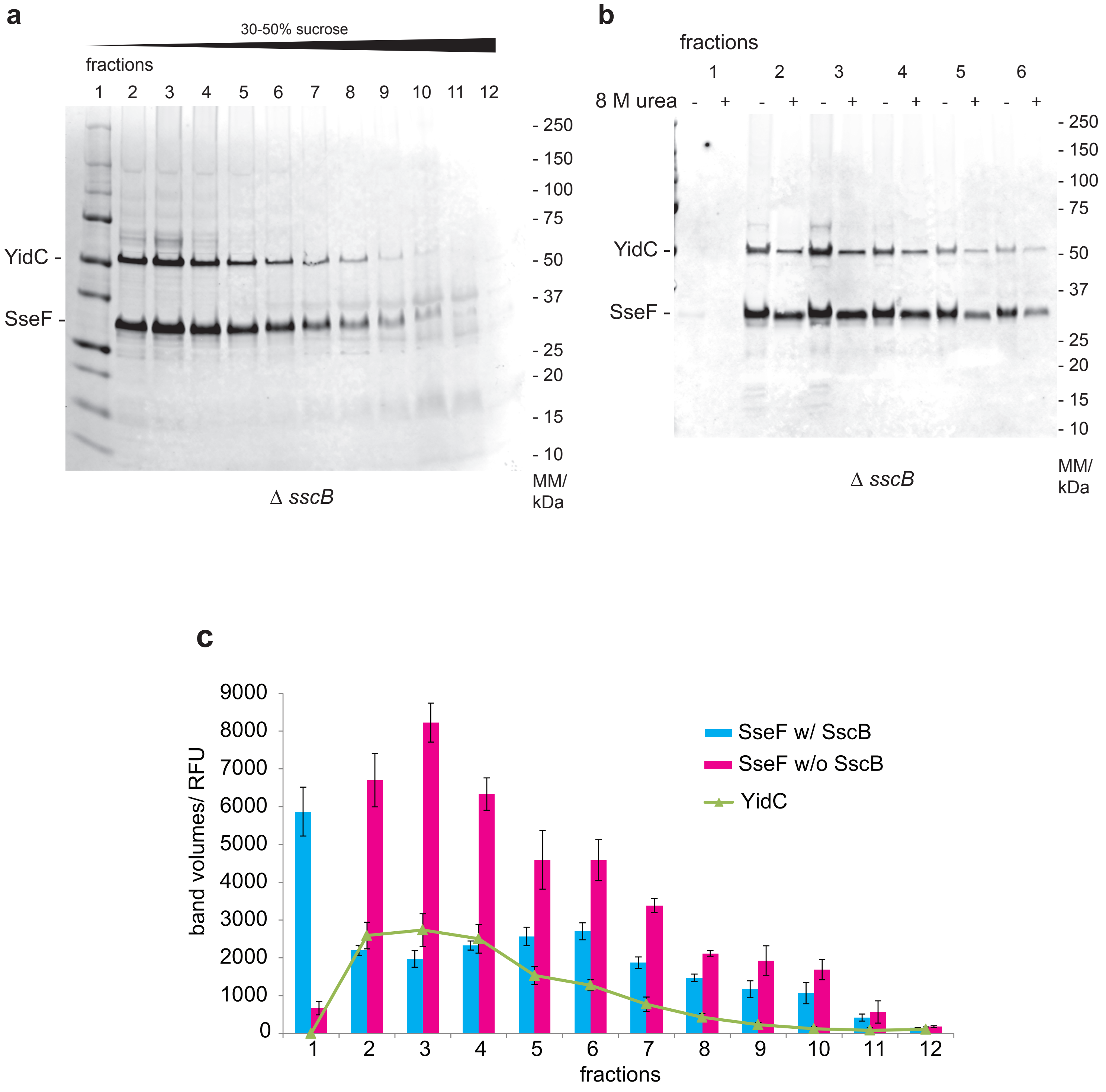

Supplementary Figure 13: SseF co-purifies with inner membrane fractions in the absence of its cognate chaperone SscB

a To assay the propensity of SseF to integrate into the inner membrane of Salmonella in the absence of its chaperone SscB, $SseF^{FLAG}$ was expressed from a low-copy number plasmid and analyzed by SDS PAGE and Western blotting of bacterial membrane fractions separated by sucrose-gradient centrifugation. Immunodetection was done with anti-FLAG and anti-YidC (inner membrane control) antibodies. Fraction 1 also contained the molecular mass standard. **b** Immunodetection of $SseF^{FLAG}$ in bacterial inner membrane fractions (#1-6) treated with and without 8M urea. Representative results of three independent experiments are shown. **c** Volumes of bands of SseF expressed with chaperone (blue; **Supplementary Fig. 4**), SseF expressed without chaperone (red) and YidC (green, representing the inner membrane) of the indicated fractions of the sucrose gradient were quantified by the Odyssey imaging system Image Studio 3.1 (Li-Cor). Representative results of three independent experiments are shown. Error bars indicate the standard deviation according to a Student's t-test (n = 3). Abbreviations: RFU: relative fluorescence units.

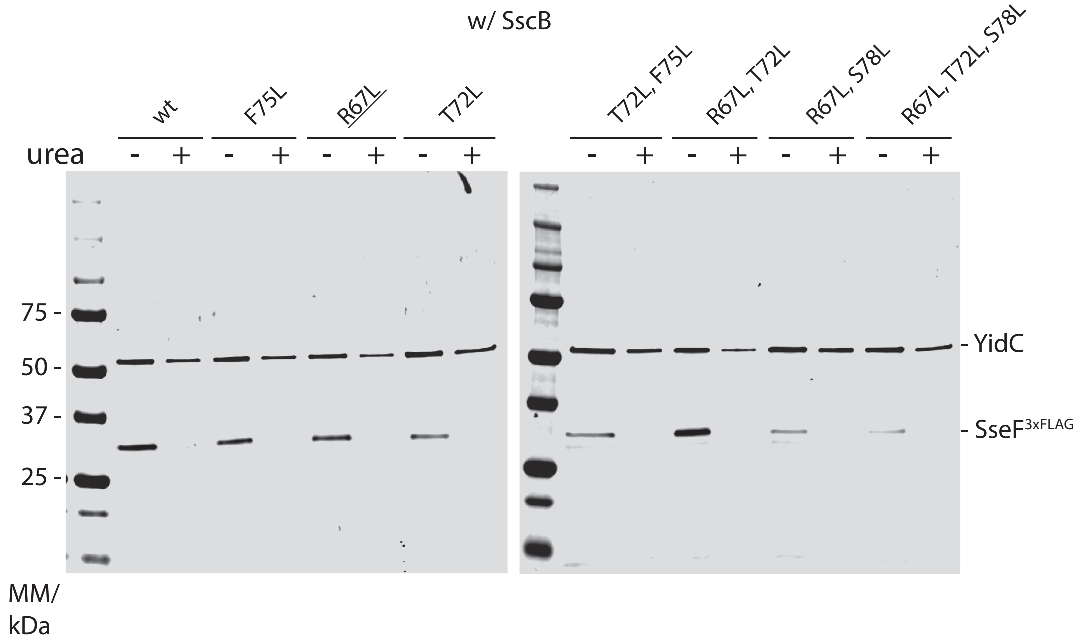

Supplementary Figure 14: Uncropped image of the Western blot shown in Fig. 4

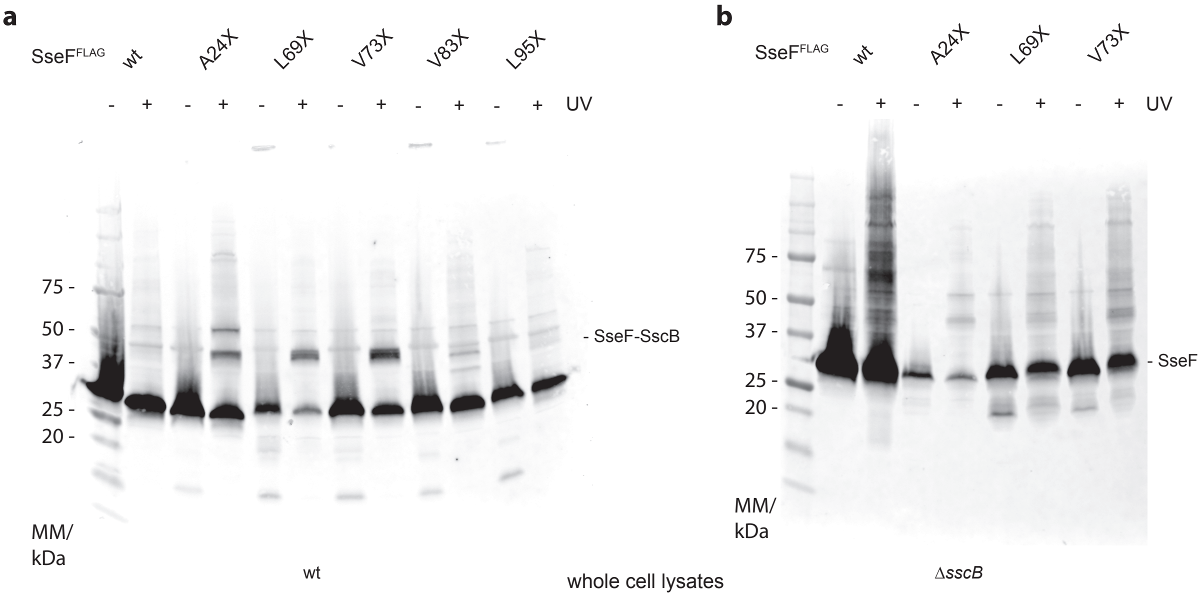

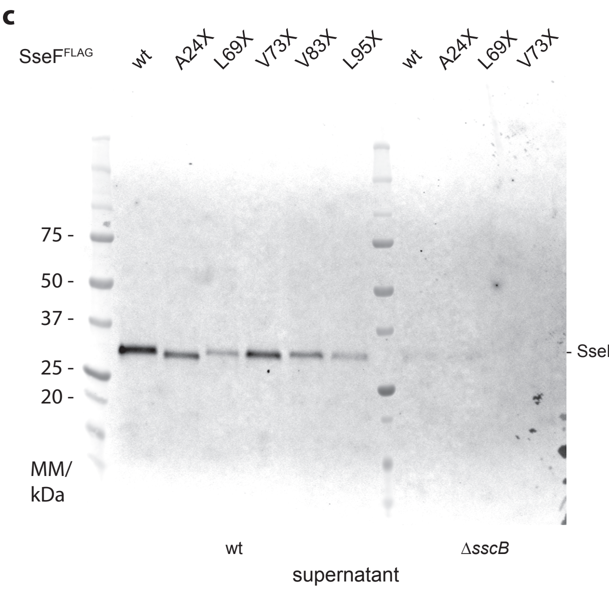

Supplementary Figure 15: Uncropped images of the Western blots shown in Fig. 5

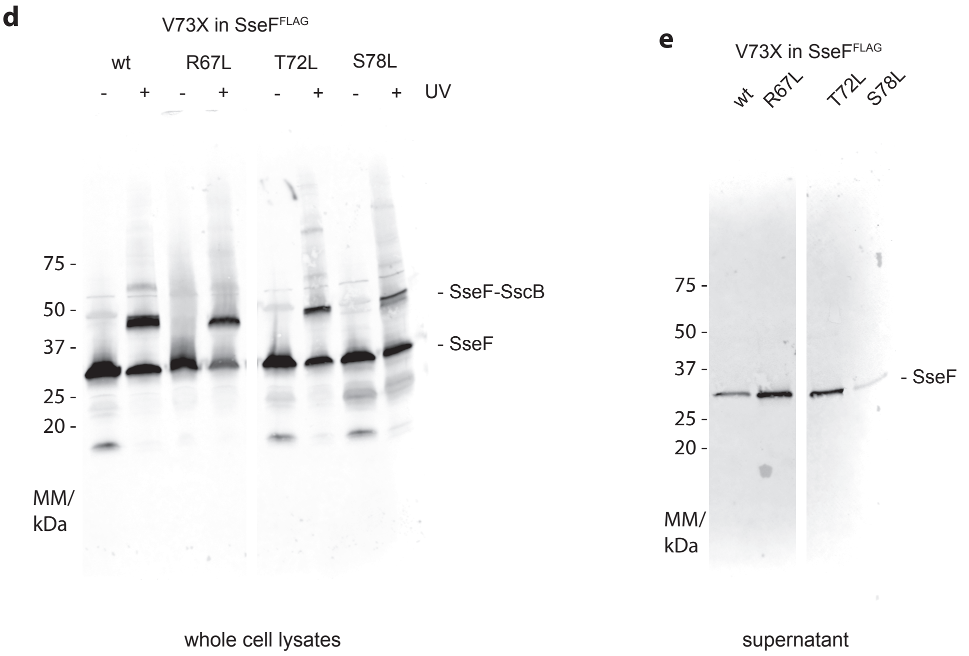

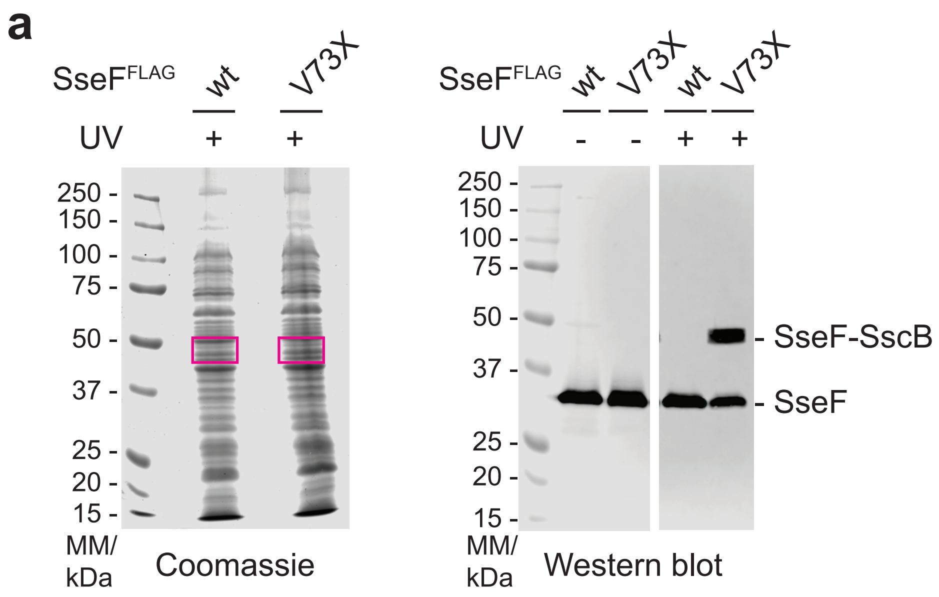

b

Proteins	MM/kDa	Peptides		Sequence coverage [%]		Intensity	
		wt	V73X	wt	V73X	wt	V73X
SscB	16.4	0	8	0	49.3	0	4.21E+08
SseF	26.7	0	3	0	20.4	0	2.48E+08

Supplementary Figure 16: Mass-spectrometric confirmation of SscB crosslinked to $\text{SseF}_{\text{V73pBpa}}$
a *Salmonella* expressing $\text{SseF}^{\text{FLAG}}$ (no pBpa control) or $\text{SseF}_{\text{V73pBpa}}{}^{\text{FLAG}}$, respectively, from a low copy number plasmid were UV-irradiated for 30 min and their protein content was subsequently analyzed by SDS PAGE and Coomassie staining (left) or Western blotting/anti FLAG immunodetection (right), respectively. Gel slices at the height of the $\text{SseF}_{\text{V73pBpa}}{}^{\text{FLAG}}$ crosslink were cut from the Coomassie-stained gel of the $\text{SseF}_{\text{V73pBpa}}{}^{\text{FLAG}}$ sample and $\text{SseF}^{\text{FLAG}}$ control, respectively, (red boxes) and analyzed by mass spectrometry. **b** The table contains the mass spectrometry results including detected peptides, sequence coverage [%] and peak intensity. A full list of LC-MS/MS identifications is provided in **Supplementary Data 2**.

Supplementary References

1. Omasits, U., Ahrens, C. H., Müller, S. & Wollscheid, B. Protter: interactive protein feature visualization and integration with experimental proteomic data. *Bioinformatics* **30,** 884–886 (2014).
2. Kim, B. H., Kim, H. G., Kim, J. S., Jang, J. I. & Park, Y. K. Analysis of functional domains present in the N-terminus of the SipB protein. *Microbiology (Reading, Engl)* **153,** 2998–3008 (2007).
3. Abrahams, G. L., Müller, P. & Hensel, M. Functional dissection of SseF, a type III effector protein involved in positioning the *Salmonella*-containing vacuole. *Traffic* **7,** 950–965 (2006).
4. Thomas, N. A., Deng, W., Baker, N., Puente, J. & Finlay, B. B. Hierarchical delivery of an essential host colonization factor in enteropathogenic *Escherichia coli*. *J Biol Chem* **282,** 29634–29645 (2007).
5. Hessa, T. *et al.* Molecular code for transmembrane-helix recognition by the Sec61 translocon. *Nature* **450,** 1026–1030 (2007).
6. Schibich, D. *et al.* Global profiling of SRP interaction with nascent polypeptides. *Nature* **536,** 219–223 (2016).
7. Fröderberg, L. *et al.* Versatility of inner membrane protein biogenesis in *Escherichia coli*. *Mol Microbiol* **47,** 1015–1027 (2003).
