# Supplementary material for: Revealing the mechanisms of membrane protein export by virulence-associated bacterial secretion systems
Source: Nat Commun. 2018 Aug 27;9:3467. doi: 10.1038/s41467-018-05969-w (PMC6110835; doi:10.1038/s41467-018-05969-w)
Supplement: Supplementary file 3 — Description of Additional Supplementary Files [file 41467_2018_5969_MOESM3_ESM.docx]

**Description of Additional Supplementary Files**

File Name: Supplementary Data 1

Description:

ΔG prediction of T3SS and T4BSS TMD-containing substrates for membrane partitioning (window 18-35, length correction ON) and SRP binding (window 12-17 length correction OFF)

File Name: Supplementary Data 2

Description:

List of proteins identified by mass spectrometry in SseFV73pBpa crosslinked band and uncrosslinked control

File Name: Supplementary Data 3

Description:

Strains, plasmids, and primers
